# US INDIAN HEALTH SERVICE: DEVELOPING A COMMUNITY OF PRIMARY CARE CHAMPIONS FOR TRIBAL ELDER CARE

**DOI:** 10.1093/geroni/igae098.0843

**Published:** 2024-12-31

**Authors:** Jolie Crowder

**Affiliations:** Indian Health Service, Rockville, Maryland, United States

## Abstract

As part of this symposium, the Indian Health Service (IHS) Alzheimer’s Grant Program reports on lessons learned during the first two pilot years of new geriatric workforce development initiatives. The IHS Alzheimer’s program partners with local IHS, Tribal, and Urban (I/T/U) health programs to address Alzheimer’s and Related Dementias (ADRD) among American Indian and Alaska Native people receiving care in the “Indian Health system.” The Indian Health Geriatric Scholars (GeriScholars) program, Geriatric Nurse Fellowship (GNF), and Project ECHO (Extension for Community Healthcare Outcomes) aim to build capability in geriatric care and care for dementia within primary care and to develop a community of primary care champions for improved care for older American Indian and Alaska Native people. Adapted from the VA Geriatric Scholars program, the Indian Health Gerischolars and Nurse Fellowship pilots provide clinicians who are the mainstay workforce in our primary care delivery system with training and mentorship. The use of the ECHO model facilitates ongoing mentorship and training and sustains a community of practice with an emphasis on those with ADRD and caregivers. The programs aim to deepen individual clinical knowledge and skills in geriatric care and then apply newfound knowledge to improve care. Local assessment of needs and priorities for improving and integrating new skills and knowledge in ongoing primary care delivery are emphasized. Initiative planning and program improvements consider unique cultural contexts and disproportionate systems and resource constraints, and are designed to work in concert with other systems-focused efforts, including Alzheimer’s Models of Care grants.

